# Persistent Vegetative State Following a Cardiac Arrest in a Patient With Preeclampsia and Posterior Reversible Encephalopathy Syndrome: A Case Report

**DOI:** 10.7759/cureus.78757

**Published:** 2025-02-09

**Authors:** Nebojsa Brezic, Aleksandar Sic, Strahinja Gligorevic

**Affiliations:** 1 Department of Anesthesiology, Resuscitation, and Critical Care, University Clinical Center of Serbia, Belgrade, SRB; 2 Department of Neurology, University of Belgrade, School of Medicine, Belgrade, SRB; 3 Department of Internal Medicine, University of Belgrade, School of Medicine, Belgrade, SRB

**Keywords:** cardiac arrest, case report, persistent vegetative state, posterior reversible encephalopathy syndrome, preeclampsia

## Abstract

We report the case of a 35-year-old female patient who presented to a tertiary referral hospital in a persistent vegetative state (PVS) following a cardiac arrest that occurred approximately one hour after an elective cesarean delivery for a postterm pregnancy complicated by preeclampsia. Magnetic resonance imaging (MRI) of the brain performed seven days after the cardiac arrest showed findings consistent with posterior reversible encephalopathy syndrome (PRES). Following transfer to our hospital, the patient was carefully evaluated, stabilized, and successfully weaned off mechanical ventilation. However, due to the lack of neurological recovery, the decision was made to transfer her to a neurorehabilitation center for further management. This case report explores the potential link between PRES and PVS.

## Introduction

Preeclampsia is a hypertensive disorder of pregnancy (HDP) that affects 5% to 7% of gravid women worldwide and remains a leading cause of maternal morbidity and mortality, contributing to over 70,000 maternal and 500,000 fetal deaths globally each year [1]. Characterized by new-onset hypertension and proteinuria or evidence of end-organ damage after 20 weeks of gestation, preeclampsia carries a risk of severe complications, including the rare yet potentially serious posterior reversible encephalopathy syndrome (PRES) [2,3]. Posterior reversible encephalopathy syndrome arises from disrupted autoregulatory mechanisms in the posterior circulation, leading to vasogenic edema and manifesting with headaches, visual disturbances, seizures, and altered consciousness [4,5]. A recent study revealed that 22.5% of hypertensive pregnant women who presented with seizures and would typically be diagnosed with eclampsia were actually found to have PRES, highlighting the fine line between PRES and HDP [6]. In certain cases, PRES can lead to long-term neurological impairment, as seen in cases of eclampsia and severe preeclampsia. This malignant PRES is characterized by radiographic findings consistent with PRES, a Glasgow Coma Scale (GCS) score of less than eight, and clinical deterioration despite standard management for intracranial hypertension [7].

Persistent vegetative state (PVS) is a disorder of consciousness in which a patient retains wakefulness but lacks awareness of themselves or their surroundings. It is characterized by the absence of purposeful behavior, language comprehension, and voluntary responses to stimuli. However, the patient may still exhibit autonomic functions such as sleep-wake cycles, cranial nerve reflexes, and spinal reflexes. The condition typically arises from brain lesions that disrupt the communication between the cerebral cortices and the thalami while sparing essential brainstem functions, with common causes including hypoxic-ischemic injury following a cardiopulmonary arrest and diffuse axonal injury resulting from trauma. Prognosis is often poor, particularly for non-traumatic causes, with less than a 1% chance of significant neurological recovery if awareness is not regained within three months [8].

## Case presentation

A 35-year-old female patient was admitted to a regional hospital for an elective cesarean delivery at 40 weeks of gestation. Aside from preeclampsia diagnosed during the third trimester of pregnancy, her past medical history was otherwise unremarkable. She was prescribed methyldopa and extended-release nifedipine following the establishment of the diagnosis, although her compliance with these medications was unknown. The pregnancy was otherwise uneventful, with frequent prenatal visits and regular obstetric follow-ups. The cesarean delivery was uneventful, resulting in the birth of a healthy newborn. Postoperatively, the patient was closely monitored in the intensive care unit (ICU) due to the elevated risk for postpartum complications. No hypertensive crises were observed peripartum. Approximately one hour after delivery, she experienced a sudden cardiac arrest. Cardiopulmonary resuscitation (CPR) was initiated promptly, and return of spontaneous circulation (ROSC) was achieved after eight minutes of advanced resuscitative efforts. Post-arrest, the patient remained comatose, with a GCS score of seven, and failed to regain full consciousness over the subsequent weeks. She required prolonged mechanical ventilation and eventually underwent a tracheostomy due to ventilator dependency.

**Figure 1:**
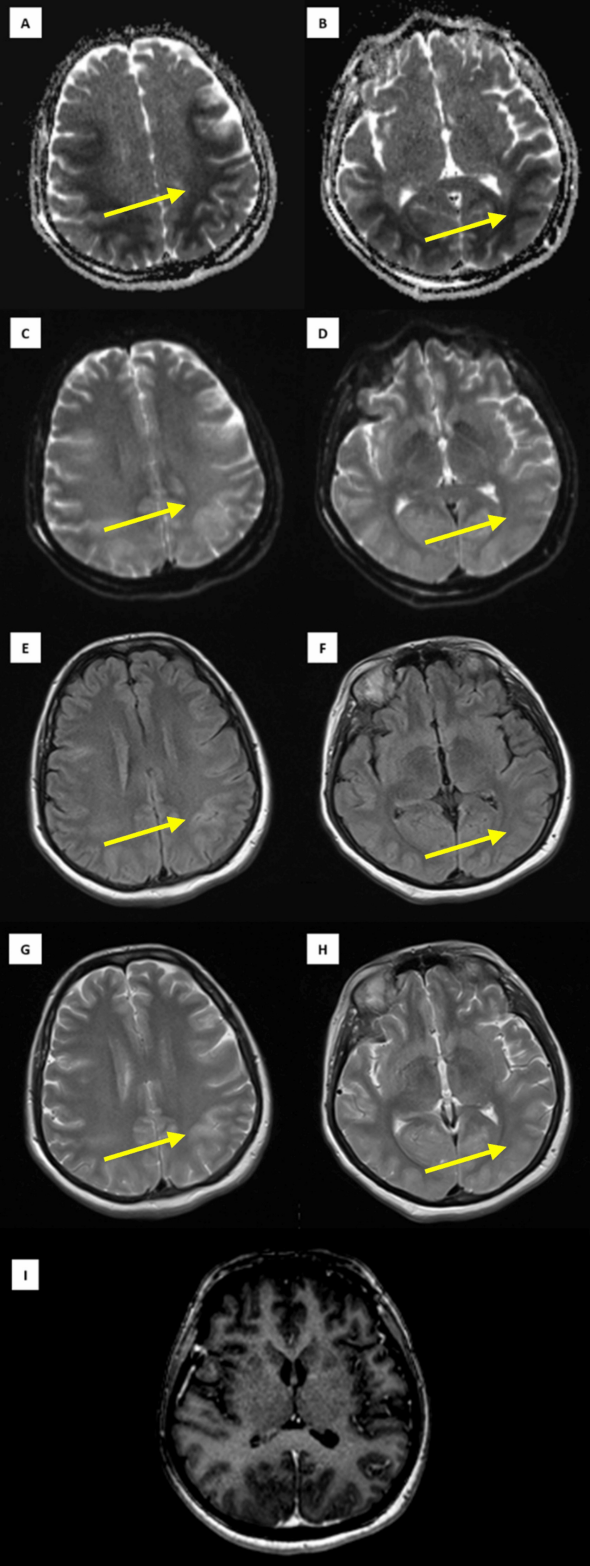
An MRI of the brain (axial) demonstrating bilateral fronto-parieto-occipital cortical and subcortical changes consistent with posterior reversible encephalopathy syndrome (yellow arrows). (A, B) Apparent diffusion coefficient (ADC) maps showing restricted diffusion in the white matter; (C, D) Corresponding bright signals on diffusion-weighted imaging (DWI); (E, F) Fluid-attenuated inversion recovery (FLAIR) images; (G, H) T2-weighted imaging (T2WI) revealing bilateral hyperintense cortical lesions; (I) Contrast-enhanced T1-weighted imaging (T1W C+) showing no postcontrast enhancement; the cortex appears diffusely expanded bilaterally, with no notable changes in the cerebellum, thalamus, basal ganglia, or brainstem.

The initial computerized tomography (CT) scan of the brain, obtained immediately after cardiac arrest, showed no signs of acute pathology. Magnetic resonance imaging (MRI) of the brain performed seven days post arrest revealed findings consistent with PRES (Figure 1). A follow-up CT scan performed four days later showed no significant structural abnormalities in the brain, while CT angiography showed no evidence of perfusion deficits.

One month after the cardiac arrest, the patient was transferred to a tertiary referral hospital for further care. On admission, she was alert with eyes spontaneously open but unable to respond to visual or vocal stimuli. She showed minimal but symmetrical defensive responses to noxious stimuli. Cranial nerve examination revealed isocoric pupils sluggishly reactive to light and intact corneal reflexes bilaterally. The right plantar reflex was positive, and the patient exhibited decorticate posturing. Physical examination showed normal muscle trophism but impaired active and passive mobility with increased bilateral muscle tone, consistent with spastic quadriparesis (hyperflexion type). Deep tendon reflexes were symmetrically heightened with pathological reflexes. Muscle strength could not be assessed. Complete muscle tone loss was noted bilaterally. The GCS was eight. The patient was admitted with a tracheal cannula and was receiving mechanical ventilation. She was transferred to the ICU for further management, where she was ultimately weaned off mechanical ventilation and received a percutaneous endoscopic gastrostomy (PEG) tube. During her ICU stay, an electroencephalogram (EEG) was obtained, which showed a flattened baseline activity with intermittent high-voltage spikes at 30 Hz, each lasting less than one second, suggesting severe electrocortical dysfunction. These findings were globally correlated with generalized myoclonus.

While in the ICU, she received clonazepam 2 mg two times a day (BID), tizanidine 2 mg three times a day (TID), amantadine 100 mg BID, and levetiracetam 500 mg BID for seven days, followed by 1,000 mg BID as prescribed by the neurologist. Despite these interventions, there was no significant neurological improvement, and the patient remained in a PVS. After 15 days in the ICU, the decision was made to transfer her to a neurorehabilitation center for advanced management and multidisciplinary care.

## Discussion

The frequency of PRES in HDP varies across studies, but a recent systematic review found that at least 50% of patients diagnosed with eclampsia and 20% of those diagnosed with severe preeclampsia had PRES on neuroimaging [9]. While PRES is commonly associated with hypertension, studies have failed to show a statistically significant difference in blood pressure between PRES and non-PRES patients with HDP [9], with only one study reporting a statistically higher diastolic blood pressure in those with PRES [10]. These results are consistent with our case report, as our patient did not experience any hypertensive crises peripartum, suggesting that additional factors may influence the development of PRES in HDP.

The association between cardiac arrest and PRES is well-documented, with multiple cases reporting PRES following cardiac arrest, even in patients with HDP [11,12]. However, the causal direction remains ambiguous in most reported cases, as no patients underwent neuroimaging prior to the cardiac arrest. In our patient, PRES was diagnosed seven days after the cardiac arrest, leaving it unclear whether it had been present beforehand as a complication of preeclampsia.

Myoclonus is a common neurological sequel of cardiac arrest and is often associated with hypoxic brain injury. However, myoclonic jerks have also been observed in patients post cardiac arrest in the absence of MRI findings consistent with hypoxia [13]. On the other hand, cases of myoclonus and even opsoclonus-myoclonus have been described in patients with extensive PRES affecting the cerebellum, even without evidence of ischemia, suggesting that PRES itself can lead to myoclonus [14,15]. Our patient exhibited clinical and EEG findings consistent with Lance-Adams syndrome, but it remains challenging to determine whether the myoclonus was hypoxic in origin or a result of PRES.

Lesions in specific brain regions are strongly associated with PVS, with the thalamus being the most frequently affected. Subcortical white matter damage is nearly universal, disrupting cortical-thalamic connections and rendering the intact cortex non-functional. While diffuse axonal injury, neocortical ischemia and/or focal damage, and intracranial hematomas might contribute to PVS, profound damage to the subcortical white matter and thalamus consistently underpins the vegetative state [16]. While the thalamus remained unaffected in our patient, there was notable involvement of both the cortical and subcortical fronto-parieto-occipital white matter. A case series by Dasari et al. (2020) documented women who suffered cardiac arrest due to hypertension in pregnancy, some of whom developed hypoxic-ischemic brain injury, progressing to PVS [17]. Our literature search revealed no clear connections between PRES and PVS, except for a neuroimaging series study that briefly described a 55-year-old female patient who had undergone an allogeneic bone marrow transplant for myelodysplastic syndrome, complicated by gastrointestinal graft-versus-host disease treated with ciclosporin. She presented with both PRES and PVS accompanied by generalized seizures. However, the study provided no additional details, and the causal relationship between the two conditions remains unclear [18]. Patients with HDP and PRES exhibit significantly greater brain involvement compared to other PRES patients, with the basal ganglia and thalamus being particularly affected [19]. In our patient, there was extensive involvement of the frontal, parietal, and occipital cortical and subcortical structures, which may ultimately explain the presentation.

Given that neuroimaging revealed no other findings that could explain the severe thalamocortical dissociation, we remain uncertain about the precise cause of PVS in our patient. Although there are no documented reports linking PRES to PVS, the extensive fronto-parieto-occipital white matter involvement, in this case, raises the possibility that PRES could have contributed to the outcome.

## Conclusions

This case highlights a complex interaction between PRES and PVS following cardiac arrest in a patient with preeclampsia. While the direct relationship between PRES and PVS remains unclear, the extensive cortical and subcortical involvement observed in this case suggests that PRES may have contributed to the development of PVS. Despite intensive neurological interventions, the patient’s lack of significant recovery underscores the challenging prognosis for patients with severe neurological impairment following cardiac arrest and complex complications like PRES. Further research is needed to explore the potential link between these two conditions and their combined impact on neurological outcomes.
